# Blood Donation Fear, Perceived Rewards, Self-Efficacy, and Intention to Return Among Whole Blood Donors in China: A Social Cognitive Perspective

**DOI:** 10.3389/fpsyg.2021.683709

**Published:** 2021-11-22

**Authors:** Zhong Li, Shuge Lei, Xiaoming Li, Yilun Zhao, Yudong Dai, Shengxuan Jin, Qiang Fu, Xubing Cai, Zhenping Lin, Xiaoming Tu

**Affiliations:** ^1^School of Health Policy and Management, Nanjing Medical University, Nanjing, China; ^2^Institute of Healthy Jiangsu Development, Nanjing Medical University, Nanjing, China; ^3^Arnold School of Public Health, University of South Carolina, Columbia, SC, United States; ^4^Nanjing Red Cross Blood Center, Nanjing, China; ^5^School of Biomedical Engineering and Informatics, Nanjing Medical University, Nanjing, China

**Keywords:** blood donation fear, perceived rewards, self-efficacy, social cognitive perspective, intention

## Abstract

With the increasing demand from aging population and seasonal blood shortage, recruiting and retaining blood donors has become an urgent issue for the blood collection centers in China. This study aims to understand intention to donate again from a social cognitive perspective among whole blood donors in China through investigating the association between the blood donation fear, perceived rewards, self-efficacy, and intention to return. A cross-sectional survey was conducted in six cities, which are geographically and socioeconomically distinct areas in Jiangsu, China. Respondents completed a self-administrated questionnaire interviewed by two well-trained medical students. A total of 191 blood donors were included in the current study. Descriptive analysis, correlation analysis, and a generalized linear regression model were used to explore the association between demographic characteristics, psychological factors, and intention to donate again. After controlling other covariates, donors with higher fear scores reported lower intention to return (*p* = 0.008). Association between self-efficacy and intention to return was statistically significant (*p* < 0.001), whereas the association between intrinsic rewards (*p* = 0.387), extrinsic rewards (*p* = 0.939), and intention to return were statistically insignificant. This study found that either intrinsic rewards or extrinsic rewards are not significantly associated with intention to donate again among whole blood donors in China, and fear is negatively associated with intention to donate again. Therefore, purposive strategies could be enacted beyond appeals to rewards and focus on the management of donors’ fear.

## Introduction

With the increasing aging population, blood demand for clinical use is rapidly increasing in China. However, amounts of eligible donors may decrease in the future, which may exacerbate the blood donation shortage (Yu et al., 2020). Under the pandemic of COVID-19, the unavailability of blood donors could also cause blood shortages (Ou-Yang et al., 2020; Wang et al., 2020). Guaranteeing an adequate and safe blood supply has become a worldwide challenge accompanying by preventable delays in treatment, even mortality and morbidity (Dzik, 2015). WHO has proposed that only voluntary blood donation can ensure the safety and availability of blood supply. Continuous recruitment and retention of blood donors is essential to maintain safety and abundant blood supply (World Health Organization, 2017). One previous study also showed that blood collected from regular voluntary blood donors is safer than blood collected from first-time donors (Glynn et al., 2000). However, 119 of the 195 countries faced with a blood supply shortage to meet their need in 2017, including China (Roberts et al., 2019).

In China, paid blood donation had caused severe public health issues and fear of blood donation among the potential donors (Adams et al., 2009). It was not until the implementation of the Blood Donation Law in 1998 that the safety of blood collection and transfusion was gradually ensured. The blood donation system in China had gradually shifted from paid blood donation to mandatory and non-remunerated donation, and finally to the fully voluntary donation in 2011 (Wang et al., 2016). The quantity and quality of blood collected from voluntary blood donors have been greatly improved (Shi et al., 2014; Yin et al., 2015). Statistics from the National Health Commission of China revealed that the voluntary blood donation rate has increased from 4.8‰ in 1998 to 11‰ in 2017 (National Health Commission of the People’s Republic of China, 2018) and just achieved the goal of 10–20‰ voluntary blood donation rate set by the WHO (World Health Organization, 2017). As mandatory blood collection and mutual blood donation canceled gradually (Shi et al., 2014), the current blood supply does not keep pace with increasing demand (Greinacher et al., 2017). The blood shortage often emerged seasonally, especially during the period of the Chinese New Year Festival, which has caused the cancelation of surgeries (Ping and Xing, 2016). Various incentive policies were enacted, such as access to free clinical blood transfusion for donors and their core families, informed recognition, and blood donation remains misread by the public. One previous study indicate that about one-third of donors was repeated donors and had a college education in 2015 in Zhejiang, China (Hu et al., 2019). One previous study in Hong Kong, China also indicated that blood donation behaviors are more likely internally motivated (Suen et al., 2020). Medical students in China donated their blood mainly from the moral responsibility of altruism (Gao and Wang, 2017). However, the association between these incentive strategies and the intention of donors to return remains unknown. Therefore, barriers and motivators related to the intention to return needs to be excavated to deal with the increasing shortage of blood collection. Mandatory donation also caused fear of blood collection or mistrust of blood collection agencies (Hu et al., 2017). First, the public’s enthusiasm was blown away by the high compensation like a few days of vacation or monetary compensation in some institutions (Yin et al., 2015). Second, the frequent donation quota has caused misunderstanding that “blood donation is harmful” and “blood was collected illegally” (Jing and Yu, 2014). Traditional Chinese Medicine also believes that it is harmful to lose blood because it is part of the body (Shi et al., 2014). In addition, recent scandals of the social welfare system and illegal monetary blood collection in Beijing in 2018 (Xinhua Net, 2018) and in Gansu in 2014 (Baidubaike, 2014) once resulted in public distrust of some non-profit organizations in China.

Historical studies have demonstrated the motivators and deterrents of blood donation behaviors and intentions, including altruism (Bednall et al., 2013), the perceived need for donation (Abolfotouh et al., 2014), self-efficacy (van Dongen et al., 2012), fear (Shaz et al., 2010; France and France, 2018; Gilchrist et al., 2019; Zucoloto et al., 2019a), and extrinsic rewards (Bednall et al., 2013). As some donors carefully weigh up the perceived benefits and costs of donating, fear has been a significant cause of rejecting blood donation again (Shaz et al., 2010; France et al., 2019). Specifically, blood-related fears, including fears for the puncture needle, blood flow throughout the container, pain during the process, etc., have become deterrents for blood donation (Moloney et al., 2017; France and France, 2018). Moreover, the fear of blood donation paraphernalia could cause failure to donate or deter retention of blood donors (Kowalsky et al., 2014). Fears may negatively impact the intention to donate (Gilchrist et al., 2019). One review revealed that both donors and non-donors reported low self-efficacy and ineffective incentives to donate when facing deterrents (Bednall et al., 2013). A negative experience of blood donation also played a vital role; however, most studies indicated that blood donation is driven by altruism and individual responsibility, which could better reflect donors’ intrinsic rewards (Bednall et al., 2013). As for perceived rewards, personal health benefits play a vital role in the intention of blood donation (Glynn et al., 2003). One study among 542 donors in Nigeria showed that medical assistance is the primary motivator, 25.8% of them thought that blood storage may help their families (Olaiya et al., 2004). In Tunisia, donors were mainly motivated by solidarity, religion, health benefits, and insurance for family (Glynn et al., 2003). Free blood usage and physical examination for repeated donation and souvenirs for the first-time and young donors were also mentioned (Sanchez et al., 2001). Occasional blood donors were motivated most by the potential blood demand (Moore, 1991). Therefore, a better understanding of the association between the blood donation intention and blood donation fear, self-efficacy, and perceived rewards could certainly inform recruitment and increase the retention of donors (Charbonneau et al., 2016). In addition, relatively few studies have considered the demographic and social-economic status factors influencing the intention to donate (Shehu et al., 2015; Lee et al., 2019). Being married had a lower chance to donate both among potential donors in Germany and the United States (Shehu et al., 2015). Education attainment level is also positively associated with the blood donation intention (Lee et al., 2019; Zucoloto et al., 2019b). Overall, research on the intention to donate again is limited among voluntary whole blood donors.

In China, current studies on the blood donation intention and behaviors focused more on exploring the demographics of blood donors (Jing and Yu, 2014; Yin et al., 2015; Ping and Xing, 2016). As the demographics could not be altered, it would be more effective to examine the social cognitive factor of the blood donation intention. However, previous studies and efforts mainly focused on the recruitment of new donors, which overlooked the retention of blood donors, which is crucial to prevent donors from lapsing and inactive (van Dongen, 2015). Under the Chinese contextual, how fear influences the intention to return and whether the perceived rewards could help boost the intention of donors to return remain unclear. To inform policymakers to enact purposive strategies, the current study aimed to examine the association between the blood donation fear, perceived rewards, and self-efficacy and the intention to return among the whole blood donors in China. The current study is based on a cross-sectional survey. Based on prior research findings, we expected that married donors are likely to report a higher intention to return (H1a); repeated donors are also likely to report a higher intention to return (H1b), so does donors with higher level of educational attainment (H1c). Moreover, we predicted that: regardless of other factors, donors with greater score in the blood fears will report a lower intention to return (H2); donors with greater score in the self-efficacy of blood donation will have a greater intention to return (H3), so does donors with greater scores of perceived rewards (H4). Together, these hypotheses assess what demographic and psychological factors independently predict the intention to return among whole blood donors.

## Materials and Methods

### Study Sites and Participants

The current study was conducted in six geographically and socioeconomically distinct areas in Jiangsu Province, China. Jiangsu, which is located in the eastern coastal area in China. Although its economic and social development is higher than the average level of China, large geographical disparities exist across different cities. Six prefecture-level cities, whose economic development varied between the southern, central, and northern Jiangsu, were randomly selected. Mobile blood collection vehicles used in these cities were selected as the recruitment sites. Before the field survey, we recruited about 20 medical students to be trained as surveyors by the local administrators and research team members. In our university, community services are required. Helping survey in this study would help students to achieve the required service hours of community services. In this way, medical students were motivated to participate the survey reliably and sustainably. Medical students were also equipped with more knowledge of blood donation and asked to approach respondents as randomly as possible, which should reduce response bias. Voluntary whole blood donors and lookers were randomly approached and asked to participate in an anonymous survey. Surveyors were instructed to answer technical questions about the survey without influencing respondents about questionnaire responses. Nurses in the mobile blood collection vehicles also help check the accordance between self-reported data and demographic characteristics from the administrative information system to help the process of quality control. It took approximately 40–60 min for the participants to complete the face-to-face survey under the help of two surveyors with mutual supervision to ensure adherence to our study protocol. About 200 donors and 300 non-donors were initially planned to be interviewed; 196 donors and 277 non-donors were successfully interviewed. Among the 196 blood donors, five refused to complete the construct of intention to return, yielding a final sample of 191 (97.4%) donors in the current study.

### Measures

The questionnaire was self-administered and consisted of the following parts: socio-demographic information, knowledge about blood donation, the intention to donate again, and psychological factors related to blood donation, such as self-efficacy, fear, and perceived rewards. To suit the local cultural context, the experts specialized in blood donation services, psychology, and other disciplines were invited to review the validity of the questionnaire. A pilot survey was conducted to ensure the feasibility of the procedure and the readability of the items for potential respondents. Exploratory and confirmatory factor analyses provide a strong initial support for the reliability and validity of inventory used in the current study (Hu et al., 2017; Li et al., 2017). Based on previous studies, the blood donation intention to return, fear, perceived rewards, and self-efficacy were measured (Hu et al., 2017; Li et al., 2017). Scores of blood donation fear, perceived rewards, self-efficacy, and intention to return were calculated by the average score of all items. The validity was assessed using Cronbach’s α with α ≥ 0.6 and ≤ 0.9 considered acceptable internal consistency.

### Outcome Variable

#### Blood Donation Intention to Return

Blood donation intention to return was measured with a three-item scale to assess the willingness of the respondents to donate blood within the next 12 months (Livitz et al., 2019). The scale measures the extent to which individuals will attempt to donate blood again within the next 12 months. It consisted of three items (e.g., “I will try to donate blood within the next year”; “I intend to donate my blood during the next 12 months”; and “How likely is it that you will donate blood in the next 12 months?”) with a 5-point Likert response from 1 = “Strongly impossible” to 5 = “Strongly possible.” This scale achieved good internal consistency validity with a high Cronbach’s α of 0.82 in the current study.

### Predictor Variables

#### Blood Donation Fear

Adapted from a previous study (Chen and Ma, 2015), a six-item scale was employed to assess participants’ fear of blood donation, including vasovagal symptoms, blood flow, needle fear, pain caused by blood donation, fear of “could not meet eligibility for blood donation” (e.g., “the needles make me be afraid”). A five-point Likert response was used, ranging from 1 = “Not at all afraid or anxious” to 5 = “Extremely afraid or anxious.” A higher score on the scale indicates a higher level of blood donation fear. A good internal consistency validity was displayed in the current study with Cronbach’s α of 0.81.

#### Perceived Rewards of Blood Donation

Perceived rewards of blood donation consisted of intrinsic and extrinsic rewards in this study. It measures in which the respondents are motivated mainly by external forces, such as personal values and responsibility, awards, and free use of blood for themselves and their core families (France et al., 2017). The construct of intrinsic rewards focused on the perceived positive biological and psychological effects from blood donation was measured with five items (e.g., “blood donation makes people feel healthy”). The construct of extrinsic rewards evaluated the perceived psychosocial benefits from blood donation with four items (e.g., “blood donation is good for making new friends”). Both of these two constructs had a five-point Likert response from 1 = “Entirely disagree” to 5 = “Entirely agree.” Cronbach’s α of the intrinsic and extrinsic rewards was 0.79 and 0.61, respectively, in the current study.

#### Self-Efficacy of Blood Donation

The six self-efficacy items were used to assess an individual’s belief of his or her confidence and perceived ability to donate their blood in various situations successfully (van Dongen, 2015). (e.g., “I can make it soon if I want to donate my blood” and “I would be capable of donating blood during the next 12 months, even if I were afraid of needles”), with a five-point Likert response from 1 = “not at all confident” to 5 = “entirely confident.” Cronbach’s α of the self-efficacy was 0.78 in the current study.

### Covariates

Age, gender, marital status, place of residence and birth, level of education attainment, monthly income, and donation history were included as control variables. The current study divided the respondents into three groups (1 = 18–24 years, 2 = 25–34 years, and 3 = ≥ 35 years). Both places of residence and birth were divided into the urban and rural group. For the marital status, respondents were categorized into being currently married and unmarried groups. For the level of educational attainment, the respondents were grouped into groups with educational level of college or above and those with educational level of high school or less. Donation history was also coded as binary variables (1 = repeated donors and 2 = first-time donors).

### Statistical Analysis

First, the Shapiro–Wilk test was used to indicate the scores of psychological factors and the intention to donate again that are distributed normally or not (*p* < 0.001). The Kruskal–Wallis equality-of-populations rank tests were used to compare the intention to donate again by sociodemographic factors to help select independent variables in the regression models with the criteria at *p* = 0.10. We then conducted the Spearman and Pearson correlation analyses to investigate the association between the sociodemographic, psychological factors, and intention to donate again (Mukaka, 2012). Given the fact that variables of intention scores were not normally distributed, a generalized linear regression model was used to explore the association between included sociodemographic and psychological factors and the intention to donate again. Dominance analysis was also conducted to identify relative importance of each predictors (Ye et al., 2015). Multicollinearity was assessed using variance inflation factors (VIFs) after an ordinary least squares regression model (*R*^2^ = 0.413, Adj *R*^2^ = 0.382) that did not indicate the presence of multicollinearity among predictors (VIF = 1.49). All the statistical procedures were performed with IBM SPSS 24.0 (IBM Corp., Armonk, NY, United States) with statistical significance at two sides (*p* < 0.05).

## Results

### Basic Characteristics

Sample characteristics were summarized (Tables 1, 2). Given the fact that the variables of age [result of the Shapiro–Wilk test: *p* (skewness) < 0.001, *p* (Kurtosis) = 0.010, *p* (joint) < 0.001], monthly income [result of the Shapiro–Wilk test: *p* (skewness) < 0.001, *p* (Kurtosis) < 0.001, and *p* (joint) < 0.001] and the intention score are not distributed normally, median (P25, P75) was used to describe the corresponding variables. Among the 191 blood donors, 88 (46.1%) were female, mean age was 30.9 ± 9.8 years with a median age of 30 years. Their median monthly income was 3,000 Chinese Yuan. More than 50% of them were born in rural areas, and 74.1% of them lived in urban areas at the time of the survey. Over half (56.7%) of the participants were unmarried. About 56% of them completed a level of college education or above. Over half (55.0%, 105 of 191) of the participants were repeated donors. Married donors reported a higher intention to donate again than those who were unmarried (*p* = 0.042). Significance was maintained when analyzed by the donation history, and repeated donors reported a higher intention to donate again than the first-time donors (*p* = 0.003).

**TABLE 1:** Population statistics of all included variables.

Variables	Mean	Median	P25	P75	SD	Min	Max	Skewness
Gender	1.46	1.00	1.00	2.00	0.50	1.00	2.00	0.16
Age	30.67	28.00	23.00	38.00	9.58	18.00	57.00	0.72
Monthly income	2846.39	3000.00	1600.00	4000.00	1963.44	0.00	10000.00	0.68
Place of residence	1.26	1.00	1.00	2.00	0.44	1.00	2.00	1.10
Place of birth	1.56	2.00	1.00	2.00	0.50	1.00	2.00	−0.24
Marital status	1.43	1.00	1.00	2.00	0.50	1.00	2.00	0.27
Educational attainment	1.44	1.00	1.00	2.00	0.50	1.00	2.00	0.24
Donation history	1.55	2.00	1.00	2.00	0.50	1.00	2.00	−0.20
Fear	2.57	2.59	2.00	3.17	0.85	1.00	5.00	0.14
Intrinsic rewards	3.96	4.00	3.60	4.60	0.70	1.80	5.00	−0.44
Extrinsic rewards	3.21	3.25	2.75	3.75	0.83	1.00	5.00	−0.02
Self-efficacy	3.94	4.00	3.50	4.33	0.62	1.67	5.00	−0.45
Intention to return	3.97	4.00	3.67	4.67	0.79	1.00	5.00	−1.09

**TABLE 2:** Comparison of intention score to donate again by demographic characteristic.

Variables	Overall *N* (column %)	Intention score median (p25,p75)	*p-*value
**Gender**			0.976
Female	88 (46.1)	4 (3.67, 4.67)	
Male	103 (53.9)	4 (3.67, 4.67)	
**Age**	28 (23, 38)		0.204
18–24	66 (34.7)	4 (3.33, 4.33)	
25–34	62 (32.6)	4 (3.33, 4.67)	
35–57	62 (32.6)	4 (3.67, 4.67)	
Monthly income*	3,000 (1,600, 4,000)	NA	NA
**Place of residence**			0.697
Urban	140 (74.1)	4 (3.67, 4.67)	
Rural	49 (25.9)	4 (3.67, 4.67)	
**Place of birth**			0.365
Urban	82 (44.1)	4 (3.67, 4.67)	
Rural	104 (55.9)	4 (3.67, 4.67)	
**Marital status**			0.042
Married	106 (56.7)	4 (3.67, 4.67)	
Unmarried	81 (43.3)	4 (3.33, 4.33)	
**Educational attainment**			0.172
College or above	103 (56.0)	4 (3.33, 4.33)	
High school or less	81 (44.0)	4 (3.67, 4.67)	
**Donation history**			0.003
Repeated donors	105 (55.0)	4 (3.33, 4.33)	
First-time donors	86 (45.0)	4 (3.67, 4.67)	

*In Chinese Yuan. p is the probability value for the comparison of intention scores by different sociodemographic groups. Because the variables of age [result of Shapiro–Wilk test: p (skewness) < 0.001, p (Kurtosis) = 0.010, p (joint) < 0.001], monthly income [result of Shapiro–Wilk test: p (skewness) < 0.001, p (Kurtosis) < 0.001, p (joint) < 0.001], and intention score are not distributed normally, median (P25, P75) was used to describe intention scores.

### Correlation Analysis

Results of bivariate correlation analysis between psychological factors and the intention to donate again were presented in Table 3. Fear is negatively associated with the intention to donate again (*r* = −0.35, *p* < 0.001), whereas intrinsic rewards (*r* = 0.46, *p* < 0.001) and self-efficacy (*r* = 0.55, *p* < 0.001) are positively associated with the intention to donate again. The association between extrinsic rewards and the intention to donate again was not statistically significant (*r* = 0.09, *p >* 0.05). Detailed bivariate relationship between all variables was presented in Table 3.

**TABLE 3:** Bivariate relationship between all included variables.

Variables	1	2	3	4	5	6	7	8	9	10	11	12	13
1. Gender	1.00												
2. Age	−0.01	1.00											
3. Monthly income	−0.16*	0.41***	1.00										
4. Place of residence	−0.02	−0.03	0.15	1.00									
5. Place of birth	0.02	−0.12	0.02**	0.43***	1.00								
6.Marital status	0.01	−0.76***	−0.42***	−0.11	0.09	1.00							
7. Educational attainment	0.05*	0.34	0.11	0.18	0.25*	−0.35***	1.00						
8. Donation history	−0.04	0.39***	0.27***	0.07	−0.06	−0.38***	0.07	1.00					
9. Fear	0.23***	0.04	0.06	−0.01	−0.11	−0.01	−0.06*	−0.01	1.00				
10. Intrinsic rewards	0.07	0.21	0.11	−0.09	−0.05	−0.21**	0.15*	0.06	−0.32***	1.00			
11. Extrinsic rewards	−0.01	0.13	0.07	0.04	−0.18	−0.10	−0.05	−0.15	−0.08	0.35***	1.00		
12. Self-efficacy	0.13	0.21***	0.01	−0.02	−0.06	−0.17*	0.09*	0.16	−0.26**	0.72***	0.18**	1.00	
13. Intention to return	−0.03	0.16	0.16	0.05	−0.03	−0.21*	0.11	0.26**	−0.35***	0.46***	0.09	0.55***	1.00

*p < 0.05, **p < 0.01, and ***p < 0.001. SD. Shapiro–Wilk tests were used to indicate the scores of fear [p (skewness) < 0.428, p (Kurtosis) = 0.365, p (joint) = 0.480], intrinsic rewards [p (skewness) < 0.018, p (Kurtosis) = 0.609, p (joint) = 0.057], extrinsic rewards [p (skewness) < 0.893, p (Kurtosis) = 0.406, p (joint) = 0.699], self-efficacy [p (skewness) = 0.014, p (Kurtosis) = 0.029, p (joint) < 0.001], and the intention to return again [p (skewness) < 0.001, p (Kurtosis) < 0.001, p (joint) < 0.001] are distributed normally or not to help us to choose appropriate methods for correlation analysis.

### Multivariate Regression

Table 4 shows the results of the generalized linear regression model. After controlling other covariates, donors with higher blood donation fear have a lower score on the intention to return (*p* = 0.008). The positive association between self-efficacy and the intention to return was statistically significant (*p* < 0.001). The association between the donation history and intention to donate again was marginally significant (*p* = 0.096). However, the association of intrinsic rewards (*p* = 0.939), extrinsic rewards (*p* = 0.939), and intention to return were not statistically significant. Weights of individual predictors were also presented to identify which predictors could explain the variance of intention to donate again most. These results support the hypothesis of H1b, H2, and H3.

**TABLE 4:** Multivariate regression models on the blood donation intention to return.

Variables	Coefficients (95% CI)	*p*-value	Weights of individual predictors
Marital status			4.0%
Married (Ref)			
Unmarried	−0.076 (−0.294, 0.142)	0.495	
Donation history			4.6%
First-time donors (Ref)			
Repeated donors	0.180 (−0.032, 0.392)	0.096	
Monthly income	0 (0, 0)	0.238	2.1%
Fear	−0.150 (−0.261, −0.039)	0.008	9.5%
Intrinsic rewards	0.097 (−0.123, 0.318)	0.387	23.5%
Extrinsic rewards	−0.005 (−0.126, 0.116)	0.939	1.3%
Self-efficacy	0.542 (0.319, 0.765)	<0.001	33.6%
Constant	1.704 (0.814, 2.593)	<0.001	

*Ref, reference group. Log likelihood = −115.45, AIC = 1.76, and BIC = −609.65. Weight of individual predictors were calculated with dominance analysis after the estimation of generalized linear model (overall fit statistic = 0.413).*

## Discussion

To the best of our knowledge, the current study was the first to examine the association between the blood donation fear, perceived rewards and self-efficacy, and the intention to return among whole blood donors in China. Our findings enriched the existing knowledge on the determinants of intention to return and informed the current strategies of recruiting and retaining blood donors under the Chinese contextual. These findings showed that the intention to return was determined not only by the self-efficacy, but also by the blood donation fear and donation history.

We found that the marital status and level of educational attainment were not associated with the intention to return. Repeated donors reported a higher intention to return than the first-time donors did. Given the fact that only 20% of the first-time donors will return again within 6 months (Ownby et al., 1999), the donation status may be violated by fear as eligible donors with higher fear will not choose to donate again (Shaz et al., 2010; Hashemi et al., 2019; Zucoloto et al., 2019a). These results reveal that strategies during the period between the first and second donation should be tailed to demographic differences and donation experience, thus addressing misconceptions and promoting the first-time donors to donate again.

Consistent with findings of previous studies (France and France, 2018; France et al., 2019; Gilchrist et al., 2019; Zucoloto et al., 2019b), blood donation fears, including fears for the needles, blood flow, pain from blood collection procedure, bad service, dizziness after donation, and being physically ineligible, are negatively associated with intention to return. The current results suggest that fear played a vital role in predicting intention to return. Therefore, extra attention to reduce adverse events, such as vasovagal reactions and fatigue, should be used (van Dongen, 2015). One previous study showed that identifying, managing, and meeting expectation of donors, especially among the young and first-time donors, could help make them donate again (Bagot et al., 2016). As the most illuminating reason for not donating blood, fear should be managed to reduce perceptions of risk of donors and to promote future donation behavior as its accompanying adverse reactions, such as physiological stress (Hoogerwerf et al., 2018). Fear may also come from inflated expectation of the risk for reaction to blood donation, indicating the importance of informing donors with accurate information about their common concerns. However, the traditional strategies have neglected the importance of reducing fear of blood donation (Bednall et al., 2013). Despite the impressive progress of ensuring the safety of blood donation and the management system of the Chinese government, the public still lack confidence or trust of the blood donation agencies, which should be urgently bridged (Smith et al., 2013; Chen and Ma, 2015). Furthermore, historic studies also pointed out that recruitment and retention of blood donors are involved with social networks and communities of donors (Smith et al., 2013; Suen et al., 2020). Therefore, on one hand, blood donation centers could build a comfortable atmosphere and improve the service to attract more potential and eligible donors. Expectations of potential donors should be managed to improve donation intention by addressing specific fears. On the other hand, local administrators should be effectively trained to organize educational campaigns, thus reducing the perceived fear and improving the public’s belief in their ability to overcome the fear.

The current study reveals that extrinsic rewards were not associated with the blood donation intention. This finding is not consistent with the results of one qualitative study in China, which found that blood donation was motivated not only by altruism, but also by a system of benefits and rewards for donors and their family members (Yu et al., 2013). Given findings of the current study were based on a limited sample size of respondents, the results should be cautiously interpreted. This result might suggest that the current incentive strategies that concentrate on extrinsic benefits may have a limited effect to motivate blood donors in China to donate blood again. Moreover, the intrinsic rewards could not well predict the intention to return, indicating the current propaganda strategies should not emphasize too much of the rewards for donors. This result was consistent with a previous study that donors who donated for altruistic reasons had no desire to receive incentives (Shaz et al., 2010; Kasraian and Maghsudlu, 2012). One study conducted among the African American also proved that rewards were the least frequent reason mentioned, including test for infectious disease, free health screening, special recognition, and gifts (Shaz et al., 2010). As perceived rewards might not help boost the blood donation intention, reducing fear and other perceived barriers, such as self-defined inability to donate blood, were more important (Gilchrist et al., 2019; Zucoloto et al., 2019b). Promotional materials should describe the concerns about fear or anxiety, what happens during the donation, including access to further information, thus boosting the intention to return. Policymakers could enact purposive strategies to educate nurses about distraction techniques, deep understanding of blood donation, and applied muscle tension (Masser et al., 2016), thus making donors feel more relaxed and less feared when donating (Schreiber et al., 2005).

In addition, our results reveal that self-efficacy is positively associated with the blood donation intention, which is consistent with the results of previous studies (Bednall et al., 2013). This result indicates that donors with high self-efficacy would be stimulated if they perceived the need to donate. The awareness of public should be raised to motivate more potential donors and to enhance the retention of current donors, especially among those who reported greater self-efficacy. The great amount of blood donation in previous public crisis, such as the Wenchuan earthquake indicated that the longstanding cultural obstacles, including “giving out blood is bad to health,” might have been gradually reduced (Wang et al., 2016). Previous studies also pointed out that self-perception of poor health, which entails low self-efficacy to donate can be solved through face-to-face interviews on their concerns and educating the potential donors appropriately, to make the potential donors more self-confident (Hyde et al., 2013). Therefore, to retain donors, blood collection centers should continue to provide accurate information to improve experience of donors.

## Conclusion

This study found that either intrinsic rewards or extrinsic rewards are not significantly associated with the intention to donate again among whole blood donors in China, whereas fear is negatively associated with the intention to donate again. This study highlights social cognitive factors that may be useful for blood collection centers to develop comprehensive strategies to recruit and retain blood donors among whole blood donors in China. Our findings have some vital implications for such efforts. First, different intention scores between first and repeated donors reveal that recruitment and retention strategies should be more targeted. Second, blood donation fear should be effectively managed, thus bridging the current gap between the services and expectations of donors. Third, the current study suggests that perceived rewards were not associated with the intention of donors to give their blood again. Future strategies remain imperative to self-efficacy of boost donors to return and ensure sustainable blood supply.

## Limitation

First, limited to financial support, this study did not select respondents from the central or western China. The results may not generalizable to other regions in China. As China is a country with multiple cultural background, the minorities were not fully sampled, which should be investigated in the future studies. Second, our study only focused on the blood donation to return without longitudinal data on the real-world donation behaviors as an outcome variable. Future study should link the intention of respondents to donate again and donation behaviors to investigate how to cluster donors into different trajectories, such as regular donors, occasional donors, and other groups, using latent class analysis model with the intention to donate again. Third, blood donors were surveyed after donating blood, which might cause overreporting of willingness to donate again.

## Data Availability Statement

The original contributions presented in the study are included in the article/supplementary material, further inquiries can be directed to the corresponding author/s.

## Ethics Statement

The studies involving human participants were reviewed and approved by the Ethics Commitment of Nanjing Medical University. The patients/participants provided their written informed consent to participate in this study.

## Author Contributions

XT, ZhoL, YZ, YD, QF, XC, and ZheL designed and performed the study. ZhoL analyzed the data and drafted the manuscript. XT, SL, XL, and SJ revised the manuscript. All authors approved the final version of the manuscript.

## Conflict of Interest

The authors declare that the research was conducted in the absence of any commercial or financial relationships that could be construed as a potential conflict of interest.

## Publisher’s Note

All claims expressed in this article are solely those of the authors and do not necessarily represent those of their affiliated organizations, or those of the publisher, the editors and the reviewers. Any product that may be evaluated in this article, or claim that may be made by its manufacturer, is not guaranteed or endorsed by the publisher.
